# Long-Term Chronic Intermittent Hypobaric Hypoxia Induces Glucose Transporter (GLUT4) Translocation Through AMP-Activated Protein Kinase (AMPK) in the Soleus Muscle in Lean Rats

**DOI:** 10.3389/fphys.2018.00799

**Published:** 2018-06-28

**Authors:** Patricia Siques, Julio Brito, Karen Flores, Stefany Ordenes, Karem Arriaza, Eduardo Pena, Fabiola León-Velarde, Ángel L. López de Pablo, M. C. Gonzalez, Silvia Arribas

**Affiliations:** ^1^Institute of Health Studies, University Arturo Prat, Iquique, Chile; ^2^Department of Biological and Physiological Sciences, Facultad de Ciencias y Filosofía/IIA, Cayetano Heredia University, Lima, Peru; ^3^Department of Physiology, Faculty of Medicine, University Autonoma of Madrid, Madrid, Spain

**Keywords:** altitude, chronic intermittent hypoxia, insulin sensitivity, GLUT4, AMPK and Akt

## Abstract

**Background:** In chronic hypoxia (CH) and short-term chronic intermittent hypoxia (CIH) exposure, glycemia and insulin levels decrease and insulin sensitivity increases, which can be explained by changes in glucose transport at skeletal muscles involving GLUT1, GLUT4, Akt, and AMPK, as well as GLUT4 translocation to cell membranes. However, during long-term CIH, there is no information regarding whether these changes occur similarly or differently than in other types of hypoxia exposure. This study evaluated the levels of AMPK and Akt and the location of GLUT4 in the soleus muscles of lean rats exposed to long-term CIH, CH, and normoxia (NX) and compared the findings.

**Methods:** Thirty male adult rats were randomly assigned to three groups: a NX (760 Torr) group (*n* = 10), a CIH group (2 days hypoxia/2 days NX; *n* = 10) and a CH group (*n* = 10). Rats were exposed to hypoxia for 30 days in a hypobaric chamber set at 428 Torr (4,600 m). Feeding (10 g daily) and fasting times were accurately controlled. Measurements included food intake (every 4 days), weight, hematocrit, hemoglobin, glycemia, serum insulin (by ELISA), and insulin sensitivity at days 0 and 30. GLUT1, GLUT4, AMPK levels and Akt activation in rat soleus muscles were determined by western blot. GLUT4 translocation was measured with confocal microscopy at day 30.

**Results:** (1) Weight loss and increases in hematocrit and hemoglobin were found in both hypoxic groups (*p* < 0.05). (2) A moderate decrease in glycemia and plasma insulin was found. (3) Insulin sensitivity was greater in the CIH group (*p* < 0.05). (4) There were no changes in GLUT1, GLUT4 levels or in Akt activation. (5) The level of activated AMPK was increased only in the CIH group (*p* < 0.05). (6) Increased GLUT4 translocation to the plasma membrane of soleus muscle cells was observed in the CIH group (*p* < 0.05).

**Conclusion:** In lean rats experiencing long-term CIH, glycemia and insulin levels decrease and insulin sensitivity increases. Interestingly, there is no increase of GLUT1 or GLUT4 levels or in Akt activation. Therefore, cellular regulation of glucose seems to primarily involve GLUT4 translocation to the cell membrane in response to hypoxia-mediated AMPK activation.

## Introduction

Glucose metabolism has been suggested to be improved under different types of hypoxic conditions. In fact, several studies have shown that under chronic hypoxia (CH) and short-term chronic intermittent hypoxia (CIH), glycemia and insulin levels decrease and insulin sensitivity increases in humans (Woolcott et al., 2015) and murine models (Chiu et al., 2004; Chen et al., 2010; Gamboa et al., 2011). However, the changes in long-term CIH have rarely been studied. The definitions of short- and long-term CIH vary. Short-term CIH includes minutes or hours of hypoxia during a brief period, while long-term CIH includes several days of hypoxia over a longer period of time (Siques et al., 2006).

Hypoxia exposure induces an adaptation process to replenish ATP in the cells that generate changes in glucose metabolism (Xia et al., 1997; Gamboa et al., 2011). There are several mechanisms involved in glucose regulation under hypoxic conditions, with the most important role played by glucose transporters (GLUTs) (Chiu et al., 2004; Castrejón et al., 2007; Manolescu et al., 2007). Cellular glucose uptake is mainly accomplished via facilitated transport mediated by a family of GLUTs: GLUT1 is responsible for basal glucose uptake, and GLUT4 is an insulin-regulated GLUT; both transporters are present in skeletal muscles (Scheepers et al., 2004). Insulin and energetic stress increase GLUT4 membrane translocation in skeletal muscles by different signaling mechanisms. Each stimulus, alone and in combination, results in the translocation of intracellular GLUT4 from vesicles to the cell membrane (Thong et al., 2005; Mackenzie and Watt, 2016). One of the mechanisms involved is the insulin pathway, which activates the PI3K/Akt pathway, acting as a metabolic sensor that responds to insulin stimulation and incorporates GLUT4 into the plasma membrane (Kohn et al., 1996). However, under stressful conditions, such as exercise or hypoxia, glucose transport in skeletal muscles occurs via the insulin-independent AMP-activated protein kinase (AMPK) pathway (Hayashi et al., 2000).

Under hypoxic conditions, the AMPK pathway participates in skeletal muscle glucose uptake. When intracellular ATP levels decrease, AMPK switches off ATP-consuming pathways and switches on alternative pathways for ATP regeneration, altering the AMP/ATP ratio (Hayashi et al., 2000; Carling, 2004). It appears that under hypoxic conditions, AMPK can increase glucose transport into muscle cells but also increases the rate of fat utilization by muscles (Fisher, 2006). In contrast, in mice with CH exposure, the Akt and AMPK pathways are not activated, and GLUT4 levels are unchanged (Gamboa et al., 2011). Studies in murine models under short-term CIH have shown an increase in AMPK and GLUT4 levels in skeletal muscles, though these results are controversial (Chiu et al., 2004; Li et al., 2015; Wang et al., 2015).

To our knowledge, information about the AMPK pathway and GLUT4 translocation under long-term CIH in rats is scarce. This new exposure model involves days at hypobaric hypoxia followed by days at normoxia (sea level) over a long period of time. Therefore, many pathophysiological aspects of this condition remain poorly understood or controversial, such as glucose homeostasis.

It is hypothesized that in lean rats exposed to this model of hypoxia (long-term CIH), which combines normoxic and hypoxic periods, the pathways involved in GLUT4 translocation in the soleus muscle are activated resulting in changes in glucose and insulin levels. Thus, an experimental study was designed to evaluate differences in the levels of AMPK, Akt and GLUT4 and the cellular location of GLUT4 in soleus muscles of lean rats exposed to long-term CIH compared to those in CH and normoxia (NX) rats.

## Materials and Methods

### Experimental Model and Study Groups

In this study, 30 male adult Wistar rats (3 months old; body weight 251.6 ± 1.9 g) were obtained from the animal facility of the Institute of Health Studies of Arturo Prat University, Iquique, Chile. The rats were placed in individual cages at a temperature of 22 ± 2°C and a circadian rhythm of 12 h of light and 12 h of dark. Feeding consisted of 10 g/day of food that contained 22.0% crude protein, 5.0% crude fat, 5.0% crude fiber, 9.0% ash, and 12% moisture (5POO^®^, LabDiet ^®^, Prolab RMH3000) and water *ad libitum*. Food intake was measured every 4 days by determining the amount of residual food. Movement inside the cage was not restricted, but no exercise was performed.

The rats were randomly distributed into three experimental groups, as follows: normobaric normoxia (NX), which served as a sea level control (*n* = 10); chronic intermittent hypobaric hypoxia (CIH), with 2 days of exposure to hypobaric hypoxia alternating with 2 days of exposure to NX (*n* = 10); and chronic hypobaric hypoxia (CH), which involved permanent exposure to hypoxia (*n* = 10). The exposure time of each group was 30 days, and the hypobaric hypoxia was simulated in a chamber at 428 Torr, which is equivalent to an altitude of 4,600 m above sea level. Chamber conditions were as follows: internal flow of 3.14 L/min of air and humidity between 21 and 30%. The time of ascension from sea level to 428 Torr was 60 min. NX rats were located in the same room at sea level (760 Torr) and housed under the same chamber conditions as the groups exposed to hypoxia. At the end of the exposure period (day 30), the rats were euthanized with an overdose of ketamine (0.9 mg/kg of weight), organs were collected, and specific variables were measured. These experiments were performed at Arturo Prat University.

The animal protocol and experimental model were in accordance with Chilean law N° 20380 regarding animal experimentation and were approved by the Research Ethics Committee of Arturo Prat University, Iquique, Chile.

### Body Weight, Glucose and Insulin Measurements

Blood extraction (1 mL) for biochemical measurements was performed after 12 h of fasting via cardiac puncture under anesthesia (0.3 mg/kg body weight). Both biochemical and physiological parameters in all the study groups were performed at day 0 (under basal normoxic conditions) and after 30 days (immediately after descending from the chamber). The hematocrit (Hct) and hemoglobin (Hb) values were measured. Serum insulin was measured using a commercial kit (Rat Insulin ELISA Kit^®^, ALPCO, Salem, VT, United States), and glucose was measured using a glucometer (CarenSensN^®^). The HOMA2 model was used to calculate the sensitivity (HOMA2%S) index with the HOMA2 calculator version 2.2 (Diabetes Trial Unit, University of Oxford), and body weight and residual food were measured every 4 days using an electronic scale (Acculab V-1200^®^, IL, United States).

### Western Blot Analysis

In this study, 100 mg of skeletal muscle was obtained from each rat. Protein extraction was started by tissue homogenization (Stir-Pak^®^, Barrington, IL, United States) with 1 mL RIPA lysis buffer, which contains a cocktail of phosphatase and protease inhibitors (4 mM PMSF, 10 μM leupeptin, 1 mM EDTA, 1 mM EGTA, 20 mM NaF, 20 mM HEPES, and 1 mM DTT). Then, the homogenates were centrifuged (5804 R Eppendorf AG^®^, Hamburg, Germany) at 12,000 rpm for 20 min at 4°C, and the supernatant was extracted. For quantification of the total protein extracted, the Bradford reaction was used (Bradford, 1976) with a BioPhotometer (Eppendorf AG^®^, Hamburg, Germany) at 590 nm, and samples were then stored at -80°C. For western blotting, the samples were previously diluted with Laemmli 2X [0.125 M Tris-HCl, 4% SDS (p/v), 20% glycerol (v/v), 0.004% bromophenol blue, 10% β-mercaptoethanol (pH 6.8)]. The proteins were separated according to their molecular weight (MW) under an electric field via sodium dodecyl sulfate-polyacrylamide gel electrophoresis (SDS-PAGE) (30% bis-acrylamide (v/v), 150 mM Tris (pH 6.8 and 8.8), 1.0% TEMED (w/v), H_2_O). Electrophoretic separation was initiated with the application of direct current to 150 V over 80 min with a power supply (PolySience^®^, EPS-300, Taipei, Taiwan, China), and the proteins were then transferred from the SDS-PAGE gel to a polyvinylidene fluoride (PVDF) membrane at 180 mA for 90 min with a semi-dry electroblotting system (OWLTM Separation systems, Panther semi-dry Electroblotters, Thomas Scientific^®^, Barrington, IL, United States).

To avoid non-specific antibody binding, the membrane was blocked with bovine serum albumin (BSA) at a concentration range of 3–5% in TBS-T solution containing 10 mM HCl, 150 mM NaCl, 0.05% Tween-20 at pH 7.4. The blocking time was 1 h at room temperature.

Once the PVDF membrane was blocked, it was incubated with the corresponding primary antibody [GLUT4 (sc-1608), GLUT1 (sc-7903) AMPKα1/2 (sc-25792), p-AMPKα1/2 (sc-101630), Akt1/2/3 (sc-8312), p-Akt1/2/3 (sc-33437), and β-actin (sc-130657)] at a dilution of 1:500 (Santa Cruz Biotechnology^®^, CA, United States) and incubated overnight at 4°C. Finally, the membrane was incubated with secondary antibodies (anti-goat and anti-rabbit antibodies, Santa Cruz Biotechnology^®^, CA, United States) at a dilution 1:2000 in 3% BSA for 1 h at room temperature and then washed with TBS-T and imaged in a dark room with a chemiluminescence kit (Chemiluminescence West Pico^®^, Super Signal Substrate, Thermo Scientific^®^, Rockford, IL, United States). The density of the bands was measured with ImageJ and normalized according to β-actin expression.

### Confocal Microscopy

The presence of GLUT4 in the plasma membrane of soleus muscle cells was determined by immunofluorescence using confocal microscopy. After euthanasia, the soleus muscle was detached completely and immersed in 4% paraformaldehyde and embedded in paraffin. Muscles were cut transversally in relation to the direction of the muscle fibers. Slices (3 μm thick) were deparaffinized and then hydrated by incubating them in xylene three times for 5 min and in 100% ethanol and then 95% two times for 10 min. Subsequently, the sections were washed with distilled water two times for 5 min. Antigens were unmasked with citrate in a pascal pot at 95°C for 20 min and then incubated in a permeabilization buffer (0.4% TRITON X-100 in PBS) for 30 min. Then, the sections were blocked with 5% BSA and incubated in 0.4% TRITON X-100 in PBS for 1.5 h. For the detection of GLUT4, the secondary antibody Alexa Fluor^®^ 647 (A-21244) was used, and the nuclei were labeled with 4,6-diamidino-2-phenylindole (DAPI). The plasma membrane was labeled with WGA (L4895, SIGMA^®^, San Luis, MO, United States). The samples were visualized in a mounting medium (Citifluor, Aname, Spain) with a Leica TCS SP2 confocal system (Leica^®^ Microsystems, Wetzlar, Germany) at University Autonoma of Madrid, Spain, using an emission wavelength of 405 nm for DAPI and an emission wavelength of 633 nm for Alexa Fluor^®^ 647 and 488 nm for WGA. Serial images were 1 μm thick (12 μm in total) and were captured with a 63x objective at a zoom factor of 1–4 in randomly chosen areas under identical conditions of brightness, contrast, and laser power for all of the experimental groups. MetaMorph^®^ image analysis software (Universal Imaging Co., United Kingdom) was used for quantification of the total number of cells and the intensity of GLUT4 fluorescence, which was used to calculate the amount of GLUT4 present in the plasma membrane by subtracting the total intensity of the cells from the intensity of the cytoplasm.

### Data Analysis

All data recorded were included into a database and analyzed using the SPSS program (IBM SPSS^®^ V.21.0^®^, Armonk, NY, United States). The normality of the variables was established by the Kolmogorov-Smirnov test, and all variables had a normal distribution. The means, standard errors (SEs) and confidence intervals (CIs) were calculated for all variables. To determine differences in the measured variables over time, in each group, a paired-sample Student’s T test was performed. To assess the magnitude of change in variables, between days 30 and 0, the means difference and 95% CIs were calculated for each variable. After obtaining these values the means differences between groups were also calculated using paired and independent sample Student’s *T* test, respectively. Equal variances were assumed according to *F* value. To establish the inter-group differences, one-way analysis of variance (ANOVA) with the least significant difference (LSD) *post hoc* test was performed. The level of significance was established at the 95% confidence level, with *p <* 0.05 being considered significant.

## Results

### General Variables

At day 30 under both hypoxic conditions (CIH and CH) rats showed an increase in Hct (*p* < 0.001) compared to the NX group, with a higher value in the CH group (*p <* 0.01) than in the CIH group. Likewise, Hb was increased in both hypoxic conditions, with the CH group showing a non-significant trend toward higher levels than in the CIH group. Body weight was lower in both hypoxia-exposed groups (CIH and CH), without a difference between these two groups. It is important to note that food intake was 100% in the CH and NX groups, whereas in the CIH group, 60% of the rats ate only 70% of the food inside the chamber during periods of hypoxia, although during periods of NX, food intake was normal in the CIH group (**Table 1**). Means difference for day 30–0 between groups: for hematocrit NX vs CIH: -18.26 (-24.33, -12.18) and body weight NX vs CIH: -24.75 (-36.03, -13.46) were found.

**Table 1:** General characteristics.

	NX	CIH	CH	CH vs CIH means difference
Hematocrit(%)				
Day 0	45.56 ± 1.17	44.54 ± 2.48	43.04 ± 1.40	
Day 30	46.30 ± 1.72	63.54 ± 1.16^∗^#†	70.58 ± 0.69^∗^#	
Mean difference 30–0	0.74 (-3.25, 4.73)	19.0 (13.81, 24.18)	27.54 (23.50, 31.57)	
				8.54 (2.43, 14.64)
Hemoglobin(mg/dL)				
Day 0	14.18 ± 0.30	14.36 ± 0.30	14.5 ± 0.19	
Day 30	15.37 ± 0.70	19.77 ± 0.59^∗^#	20.64 ± 0.36^∗^#	
Means difference 30–0	1.19 (-0.76, 3.14)	5.41 (3.90, 6.91)	6.14 (5.04, 7.23)	
				1.04 (-0.92, 3.00)
Body weight (g)				
Day 0	251.80 ± 1.9	251.09 ± 2.8	251.50 ± 1.1	
Day 30	260.80 ± 3.8	235.40 ± 5.8^∗^#	233.10 ± 2.9^∗^#	
Means difference 30–0	9.08 (2.74, 15.41)	-15.67 (-26.03, -5.30)	-18.37 (-25.19, -11.54)	
				-2.70 (-14.22, 8.82)
Food intake (g)				
Day 0	10.0 ± 0.0	10.0 ± 0.0	10.0 ± 0.0	
Day 30	10.0 ± 0.0	7.8 ± 0.79^∗^#†	10.0 ± 0.0	
Means difference 30-0	–	-2.15 (-3.94, -0.35)	–	
				2.15 (0.48, 3.81)

*Variables measured in the normoxic group (NX; *n* = 10), chronic intermittent hypoxia group (CIH; *n* = 10), and chronic hypoxia group (CH; *n* = 10). Values are means (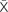) ± standard error (SEs) for hematocrit, hemoglobin, body weight and food intake; measured at day 0 and 30. ^∗^*p* < 0.001: hypoxia-exposed group vs NX; †*p* < 0.01 : CIH vs CH; #*p* < 0.001 : day 0 vs day 30. Means difference and confidence intervals (95% CI) between days 30 and 0 for each variable are shown. Means difference and confidence intervals (95% CI) of these changes between groups CH vs. CIH (days 30 to 0) are also presented.*

### Glycemia, Insulin and HOMA2%S

**FIGURE 1:**
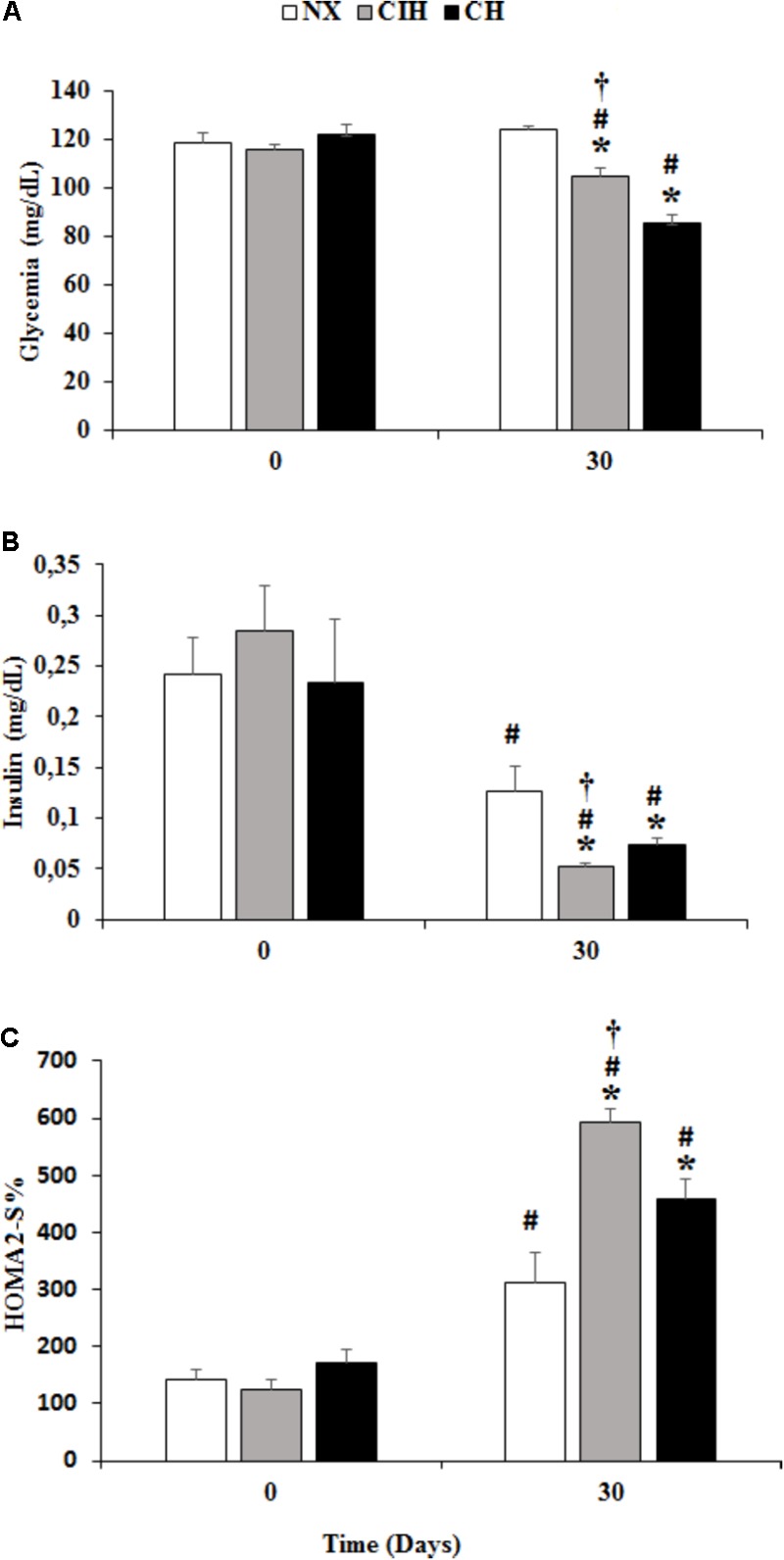
Biochemical variables. Measured in the normoxic group (NX; *n* = 10), chronic intermittent hypoxia group (CIH; *n* = 10), and chronic hypoxia group (CH; *n* = 10). Comparisons of **(A)** glycemia, **(B)** insulin level and **(C)** homeostatic model assessment 2 values (HOMA2%S) at day 0 and 30. Values are means (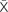) ± standard error (SE). ^∗^*p* < 0.001: hypoxia-exposed group vs. NX; ^†^*p* < 0.05: CIH vs. CH; ^#^*p* < 0.001: day 0 vs. day 30.

Both hypoxia-exposed groups (CIH and CH) exhibited a decrease in blood glucose levels at day 30 and CH showed levels lower than those in the CIH group (*p* < 0.05). There was a decrease in serum insulin levels under both hypoxic conditions, but unexpectedly, the level of insulin was lower in the CIH group than in the CH group (*p* < 0.05). The insulin sensitivity index (HOMA2%S) increased more in the CIH group than in the NX and CH groups (*p* < 0.05), and the difference was proportional to the insulin level (**Figure 1**).

### Protein Measurements: GLUTs, AMPK and Akt

**FIGURE 2:**
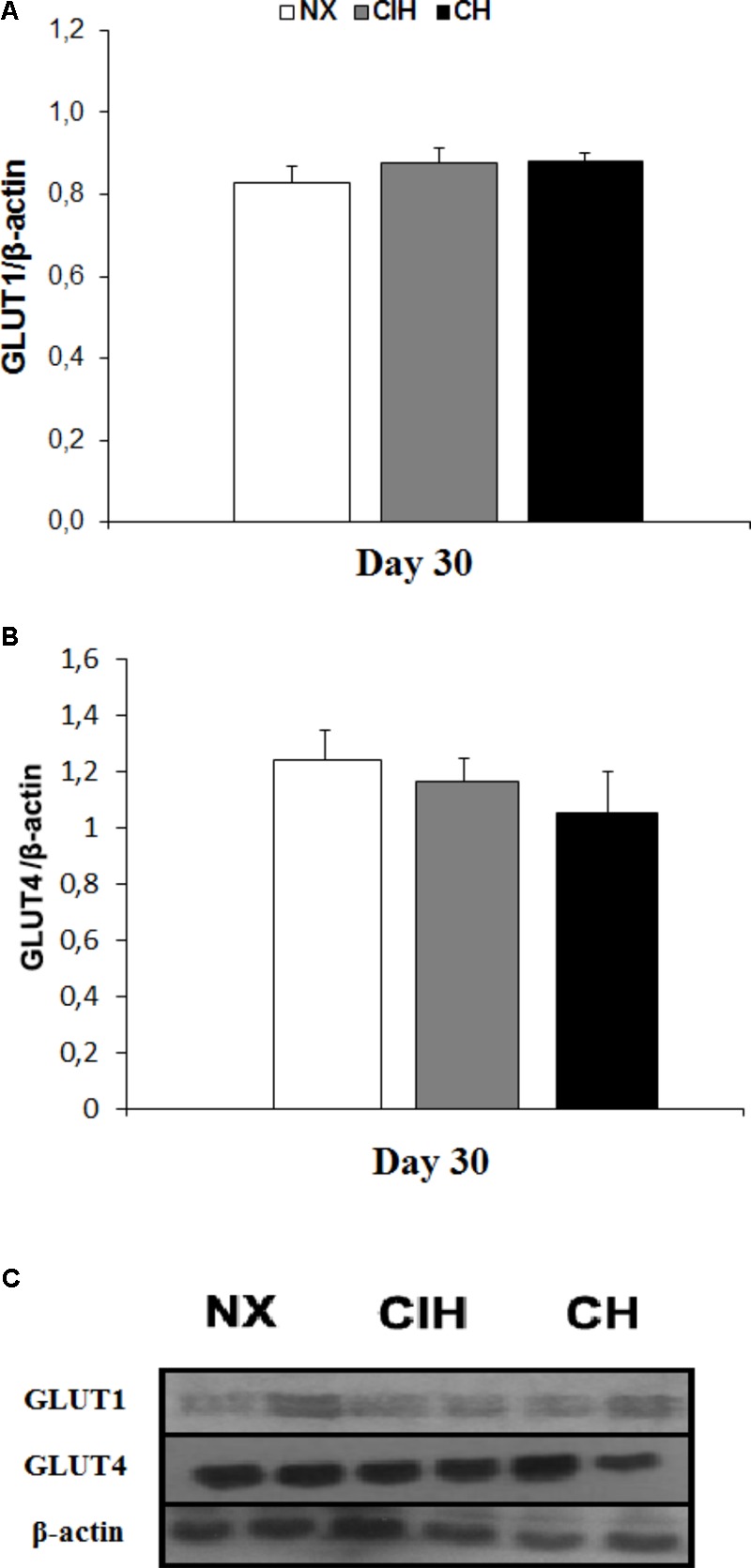
Proteins expressions levels of glucose transporters. GLUT1 and GLUT4 protein levels in soleus muscles of lean rats in the normoxic group (NX; *n* = 10), chronic intermittent hypoxia group (CIH; *n* = 10), and chronic hypoxia group (CH; *n* = 10). **(A)** GLUT1 protein levels, normalized by β-actin; **(B)** GLUT4 protein levels, normalized by β-actin; and **(C)** representative bands for GLUT1, GLUT4 and β-actin. Values in **(A)** and **(B)** are means (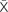) ± standard errors (SEs).

**FIGURE 3:**
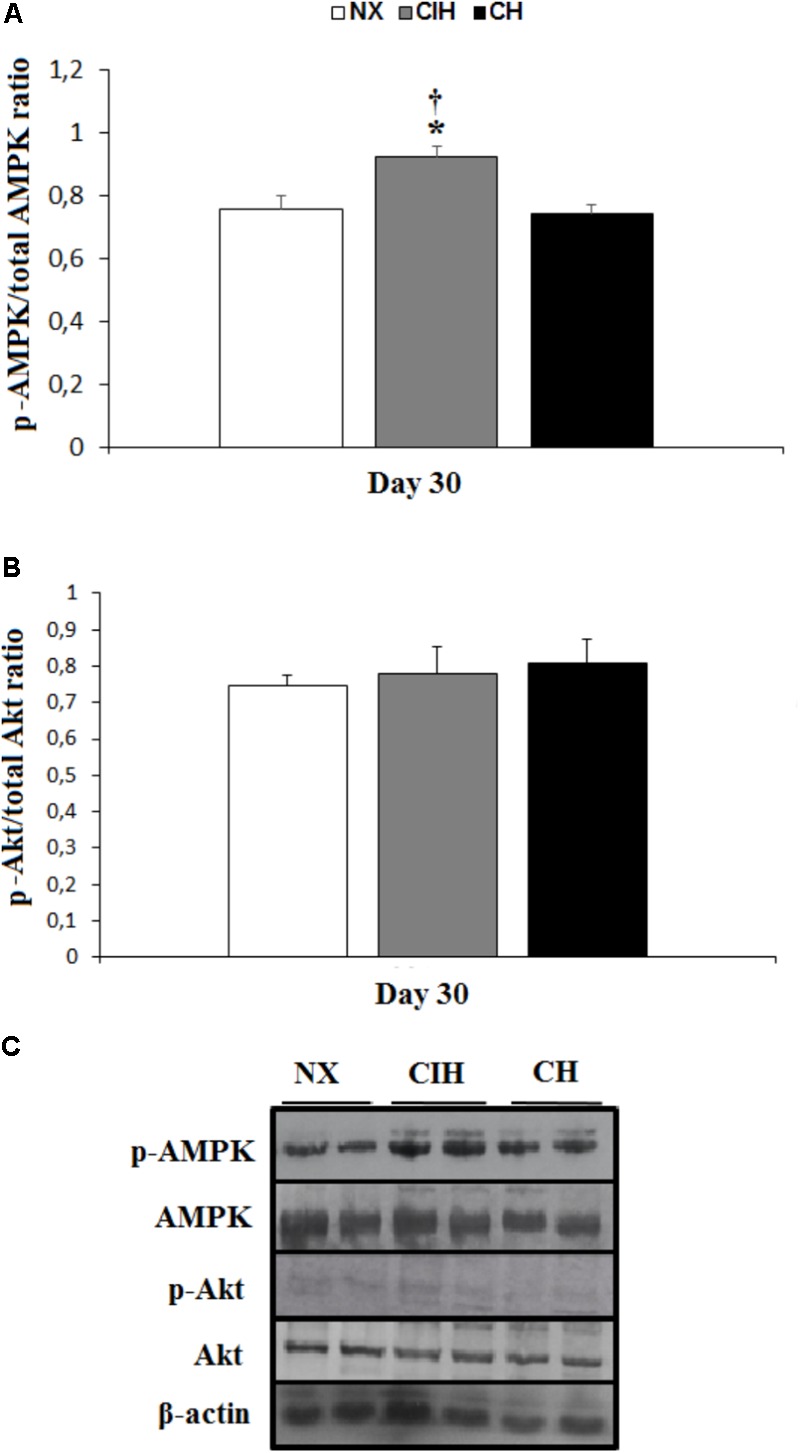
Activation of AMPK and Akt. Measured in the soleus muscles of lean rats in the normoxic group (NX; *n* = 10), chronic intermittent hypoxia group (CIH; *n* = 10), and chronic hypoxia group (CH; *n* = 10). **(A)** Comparison of p-AMPK/total AMPK ratio, normalized by β-actin; **(B)** comparison of p-Akt/total Akt ratio, normalized by β-actin; and **(C)** representative bands for activated AMPK (p-AMPK), total AMPK, activated Akt (p-Akt), total Akt and β-actin. Values in **(A)** and **(B)** are means (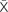) ± standard errors (SEs).^∗^*p* < 0.05: hypoxia-exposed group vs. NX; ^†^*p* < 0.05: CIH vs. CH.

The protein expression of GLUT1 and 4 was not different among groups (**Figures 2A–C**). AMPK activation, measured as the p-AMPK/total AMPK ratio, surprisingly, showed an increase in the CIH group (*p <* 0.05), whereas the CH and NX groups showed no differences (**Figures 3A,C**). Conversely, Akt activation, measured as the p-Akt/total Akt ratio, showed no difference among the studied groups (**Figures 3B,C**).

### GLUT4 Translocation

**FIGURE 4:**
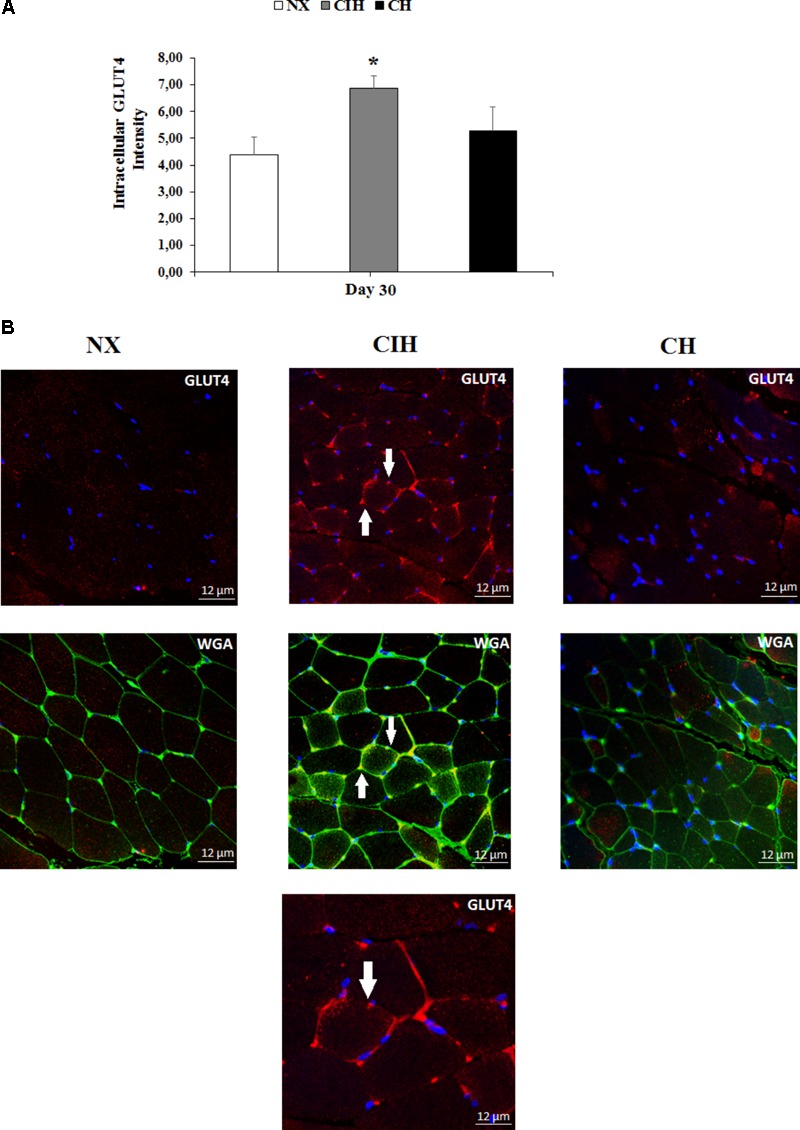
Comparison of GLUT4 location. Measured in the normoxic group (NX; *n* = 10), chronic intermittent hypoxia group (CIH; *n* = 10), and chronic hypoxia group (CH; *n* = 10). **(A)** Quantitative analysis of the intensity of GLUT4 in soleus muscles from all groups and **(B)** representative examples GLUT4 measurements obtained by confocal microscopy (x63, zoom 1). Values in **(A)** are means (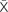) ± standard errors (SEs). ^∗^*p* < 0.05: hypoxia-exposed group vs NX. White arrow indicates GLUT4 localized in the plasma membrane of muscle. DAPI staining (first row); WGA staining (second row) and an amplified CIH cell at the third row (x63, zoom 2).

It is worth noting that the results showed a remarkable increase in the translocation of GLUT4 from vesicles to the plasma membrane in rat soleus muscles only in the CIH group (*p* < 0.05) (**Figure 4A**). These differences are also shown in representative images (**Figure 4B**).

## Discussion

This research in lean rats exposed to long-term CIH showed the following with respect to the CH and NX groups: (1) different patterns in glucose regulation, (2) lower blood glucose and plasma insulin levels and an increase in insulin sensitivity, (3) hypoxia-induced AMPK pathway activation, but not insulin-dependent Akt pathway activation and (4) an increase in GLUT4 translocation to plasma membranes in rat soleus muscle cells.

### General Findings

As expected in this model, Hb and Hct increased in the CIH group but to a lesser extent than in the CH group. These results are in agreement with those of other studies using this model, both in humans (Richalet et al., 2002) and rats (Siques et al., 2006; Brito et al., 2015). The body weight decreases observed in this study are also in agreement with previous reports in rats (Siques et al., 2006; Lüneburg et al., 2016). This latter effect could have ancillary influences on glucose metabolism, since body weight is highly correlated with insulin sensitivity (Evans et al., 1984). Nevertheless, high-altitude-induced CH leads to an inexorable loss of skeletal muscle mass as a consequence of an increase in protein degradation, which could explain the weight loss observed in this study (Chaudhary et al., 2012). Under CIH, this loss could also be attributed to a loss of appetite in rats (Bigard et al., 1996), as rats left some food uneaten during hypoxia exposure in the current study. Therefore, both mechanisms could contribute to the effect.

### Blood Glucose and Serum Insulin Levels

Previous altitude-based studies have shown that glucose homeostasis is influenced by acute or CH exposure in humans (Brooks et al., 1991; Kelly et al., 2010) and by CH exposure in mice (Gamboa et al., 2011). The current study shows that under long-term CIH, rats show improved glucose uptake, lower fasting glucose and insulin levels and increased insulin sensitivity, which also would occur under other types of hypoxia. Thus, the same phenomena have been shown in rats under short-term CIH, normobaric hypoxia and hypoxic regimen lasting hours (Chiu et al., 2004; Chen et al., 2011; Faramoushi et al., 2016), excluding obstructive sleep apnea syndrome studied in humans (Kim et al., 2013) and mice (Thomas et al., 2017). Although, controversially, some reports show no changes in glucose metabolism in rats exposed to CIH (Wang et al., 2015; Li et al., 2016). Likewise, exposure to cyclic hypobaric hypoxia in humans for 10 weeks demonstrated a decrease in glucose but no influence on insulin (Marquez et al., 2013). When drawing conclusions and making comparisons from some of the differences found in the literature regarding these effects, it must be considered that the regimen and the degree of hypoxia will lead to variation in the results (Debevec and Millet, 1985); however, most of these studies tend to agree in showing that under hypoxia, glucose regulation is improved in both rats and humans (Sawhney et al., 1986; Larsen et al., 1997; Woolcott et al., 2015; Sacramento et al., 2016). Moreover, this glucose improvement under different regimens of hypoxia would take some time and reach a plateau in the long term, resulting in rather normal value as observed in human studies (Woolcott et al., 2015; Żebrowska et al., 2018) and in rats (Chen et al., 2016). Therefore, the improved glucose regulation in rats under long-term CIH in this study supports the contribution of hypoxia to these phenomena and, to our knowledge, has not been previously reported.

### GLUT, AMPK and Akt in Soleus Muscle

Skeletal muscle is the most important regulator of glucose homeostasis and uptake (Mas et al., 2006; Gamboa et al., 2011). Glucose uptake in skeletal muscle is normally regulated by insulin-related pathways, where Akt can play a role as an insulin-stimulated signal leading to GLUT4 translocation to the cell membrane under physiological conditions (Huang and Czech, 2007). However, under exercise- and hypoxia-induced stress, the AMP/ATP ratio is increased, activating an alternative pathway: insulin-independent AMPK signaling (Hayashi et al., 2000).

It is well known that skeletal muscle has two main GLUT transporters: the constitutive GLUT1 and the insulin-dependent GLUT4, with the latter being the most relevant. In acute exposure to hypoxia GLUT4 levels increase in rats (Dill et al., 2001), and obese rats (Zucker) under short-term CIH and in response to altitude training showed increased GLUT4 levels (Chen et al., 2011), whereas under CH, no increase in GLUT4 occurs in rats and mice (Chiu et al., 2004; Gamboa et al., 2011). According to our results from long-term CIH exposure, both GLUT1 and GLUT4 levels showed no changes, similarly to what has been reported under CH. However, there was an increase in intracellular translocation of GLUT4 to the membrane. This trafficking could be considered as a compensatory mechanism to increase glucose uptake instead the increasing protein levels (Chen et al., 2008; Jørgensen et al., 2009; Gamboa et al., 2011). Interestingly, this translocation has not been previously reported in a long-term CIH model.

Additionally, Gamboa et al. (2011) found that in normobaric CH (during fasting), no activation of the Akt pathway occurs, which is consistent with the results of this study. Likewise, controversial findings indicating that chronic stress induced by reactive oxygen species (ROS) can also decrease the stimulatory effect of insulin on the Akt pathway and that glucose uptake could be mediated by the intrinsic activity of GLUT4 under hypoxic conditions have been reported (Ding et al., 2016). A role for ROS in the effect of insulin under hypoxia is a promising theory because high levels of ROS have been consistently found in humans with both acute and chronic exposure to hypobaric hypoxia (Jefferson et al., 2004) and in rats exposed to long-term CIH (Siques et al., 2014; Lüneburg et al., 2016).

The increase in GLUT4 translocation seen in this study could be explained on the basis of other pathways involved in the regulation of plasma glucose levels under energetic stress, including the AMPK pathway, as previously reported (Bradley et al., 2015). Activated AMPK acts downstream by phosphorylating and activating AS160 (Akt substrate 160), which is the main regulatory protein of the trafficking of intracellular GLUT4 (Kramer et al., 2006). It has also been shown that activated AMPK can increase the sensitivity to insulin in rats (Fisher et al., 2002). Our results support a role of AMPK but not of Akt, as the activation of AMPK was found to be increased under long-term CIH. Moreover, recent studies suggest that higher levels of insulin downregulate AMPK activity via Ser485/491 phosphorylation of the AMPK-α subunit. In this case, a lower blood insulin concentration might induce AMPK signal activation in rats (Kido et al., 2017), which would be another way of increasing AMPK activation; however, this idea needs further experimental support.

It has been observed that AMPK is activated in mice exposed to acute hypoxia (Viganò et al., 2011). This could explain the greater level of activated AMPK under CIH than under CH because, as described previously, this model involves intermittent and acute episodes of hypoxia, which results in a turn-on–turn-off regime for biological responses (Powell and Garcia, 2000). In this context, under CIH and during hypoxic training in humans, HIF-1α has been observed to be upregulated (Hoppeler and Vogt, 2001), and HIF-1α accumulation has been reported in muscle cell culture (Kubis et al., 2005). Moreover, HIF-1α could have a critical role in maintaining the GLUT4 transporter translocation in skeletal muscle cells (Sakagami et al., 2014). Since AMPK activation is consistent with the increased translocation of GLUT4 observed in this study, it could be surmised that this effect in the soleus muscle is mediated via upregulation of p-AMPK and that p-Akt does not play a role under the conditions studied here. Thus, the increases in translocation of GLUT4 to the cell membrane and AMPK activation in long-term CIH are novel findings.

Interestingly, age could play a role in GLUT regulation. Xia et al. (1997) showed that adult rats under CH exposure show slight increases in GLUT protein expression, whereas immature rats show great increases because immature tissues are more sensitive to oxygen deprivation. This latter report is almost coincident with our results regarding scarce or no GLUT4 protein increase in adult rats, although the current study is in CIH. Thus, this current study might give support to the hypothesis that in adult rats, GLUT regulation would occur not at the protein level but by GLUT translocation, resulting in increased glucose utilization.

This study in lean rats suggests that long-term CIH might have a beneficial effect in improving insulin sensitivity and glucose tolerance as has been suggested for rats exposed to short-term CIH lasting hours (Tian et al., 2016). However, several considerations must be taken into account: the hypoxia regimen and exposure duration (Xia et al., 1997); the existence of several confounding factors such as vitamin D, pollution, ozone, and diet (Woolcott et al., 2015); the differences in response to hypoxia among rat’s strain where Wistar is more intolerant to altitude (Ou and Smith, 1983; Hayward et al., 1999); and whether results from animal models can be fully extrapolated to clinical settings. Additionally, it is important to contrast the present findings with those from another model of CIH, i.e., obstructive sleep apnea syndrome, where the opposite metabolic patterns occur (Kim et al., 2013; Thomas et al., 2017).

This study has some limitations, such as the use of a very specific experimental animal model (lab rats with long-term CIH) that is more sensitive to hypoxia than human beings and with strict diet and environmental control, which increases the difficulty of comparing different CIH regimens and could prevent a direct translation of these findings into clinical or occupational health. However, this rat species was chosen, due to its known hypoxic intolerance, to assess the maximal effects of hypoxia and to perform preliminary molecular studies that would face ethical and logistic difficulties in humans. Another limitation is the difficulty of comparison given the wide variety of regimes, species, models, and the scarce reports on long-term CIH. However, this latter issue makes our results novel. Therefore, this study may contribute to the understanding of glucose metabolism in long-term CIH, which is poorly understood, and may provide directions for future research in animals and humans.

## Conclusions

Lean rats exposed to long-term CIH show a decrease in glycemia and insulin, along with an increase in insulin sensitivity compared to normoxic exposure. Interestingly, there is no increase in the levels of glucose transporter proteins GLUT1 or GLUT4 nor in the level of activated Akt. Therefore, glucose cell regulation and the relative hypoglycemia observed seem to be primarily a result of increased GLUT4 translocation to the cell membrane elicited by hypoxia-mediated AMPK activation.

## Author Contributions

PS, JB, KF, and SO conceived and designed the study, performed the experiments, analyzed and interpreted the data, drafted the manuscript, critically revised important intellectual content in the manuscript, and provided overall supervision. FL-V assisted in critical decisions and revision. KA, EP, FL-V, ÁLdP, MG, and SA contributed to the interpretation of the results and critical revisions of the manuscript. All authors approved the final manuscripts and agreed to be accountable for all aspects of the work.

## Conflict of Interest Statement

The authors declare that the research was conducted in the absence of any commercial or financial relationships that could be construed as a potential conflict of interest.
